# Video-assisted thoracoscopic surgery in children-experience of two pediatric hospitals

**DOI:** 10.36416/1806-3756/e20250111

**Published:** 2026-11-09

**Authors:** Cláudio D’Elia, Brunna de Paulo Santana, Luísa Silva Sant’Anna, Marcelo Ribeiro Mariano Holanda Duarte

**Affiliations:** 1. Centro Pediátrico da Lagoa, Grupo Prontobaby, Rio de Janeiro (RJ) Brasil.; 2. Grupo Prontobaby, Rio de Janeiro (RJ) Brasil.; 3. Escola de Medicina Souza Marques, Faculdade Souza Marques, Rio de Janeiro (RJ) Brasil.

**Keywords:** Thoracic surgery, video-assisted, Child, Lung diseases, Empyema, Thoracentesis, Thoracotomy

## Abstract

**Objective:**

To describe the indications, complications, and outcomes of minimally invasive video-assisted thoracoscopic surgery (VATS) in children by reviewing our institutional experience with VATS and comparing it with the existing literature.

**Methods:**

We conducted a retrospective observational study of 75 patients in the 1-month to 18-year age bracket undergoing VATS between February of 2020 and July of 2024 at either of two pediatric hospitals. Data were collected from medical records and analyzed for demographic and clinical variables, including sex, age, symptoms, underlying diseases, procedural indications, prior chest drainage, duration of hospitalization before VATS, culture results, postoperative chest tube duration, length of hospital stay, complications, and perioperative mortality.

**Results:**

Pleuropulmonary infections, predominantly empyema, accounted for most of the cases (89.3%). Primary VATS (without prior chest tube drainage) was performed in 26% of the patients with empyema. In cases of empyema, performing VATS within seven days of admission was not associated with a shorter postoperative chest tube duration when compared with a delayed intervention (10.8 days vs. 12.8 days; p = 0.511). However, the mean total hospital stay was significantly shorter in the early VATS group (25.5 days vs. 36.6 days; p < 0.001). The overall complication rate was 21.3%, with bleeding and subcutaneous emphysema being the most common. There were no conversions to thoracotomy and no perioperative mortality.

**Conclusions:**

Our study confirms that VATS is a safe and effective approach for managing thoracic conditions in children, particularly when performed by an experienced team. Further research is warranted to refine the timing and indications for VATS in the pediatric population.

## INTRODUCTION

The initial application of video-assisted thoracoscopic surgery (VATS) in the early twentieth century was for diagnosing and treating pleural disorders. However, its use declined with the advent of antibiotics and a concurrent lack of technological progress. 

It was not until the early 1990s, following advances in imaging techniques, surgical instruments, and anesthetic strategies, that VATS rekindled interest among thoracic and pediatric surgeons.^1^ ^,^ ^2^ Over the subsequent decades, these developments improved the safety and efficacy of VATS, significantly expanding its surgical indications and enabling its application in smaller and critically ill children. 

The first thoracoscopic interventions in children were performed in North America in the 1970s. At that time, the scope of procedures was primarily limited to biopsy, decortication, and deroofing of pulmonary cysts.^3^ A significant milestone was reached in 2000, when Rothenberg reported the first pediatric thoracoscopic lobectomy.^4^

VATS is recognized as a less invasive alternative to open surgery and may lead to superior outcomes. It has been associated with reduced hospital stays, less postoperative pain, improved cosmetic results, faster recovery, and fewer complications.^5^ ^-^ ^7^ For pediatric patients with parapneumonic effusions and empyema, VATS has proven to be a successful treatment. Nevertheless, debate persists regarding the optimal timing for its use, and it remains uncertain whether VATS should be the first-line procedure for children with pleural collections.^8^

As with any surgery, VATS can result in complications such as excessive bleeding, persistent air leaks, pneumothorax, subcutaneous emphysema, surgical site infections, intraoperative oxygen desaturation, and limited space for extracting intrathoracic samples.^9^ ^,^ ^10^

To achieve optimal outcomes, VATS requires a highly skilled and proficient team, including anesthesiologists, surgeons, and perioperative nurses. The expertise of the team directly affects critical aspects of the procedure, including accuracy, ergonomics, patient safety, and operative time. 

The objective of the present study was to describe our experience with minimally invasive VATS in children, as well as to analyze the indications, complications, and outcomes of VATS in this population. 

## METHODS

We conducted a retrospective, descriptive observational study based on a chart review of all patients who underwent VATS between February of 2020 and July of 2024 at either of two hospitals within the same pediatric health care network in the city of Rio de Janeiro, Brazil. Combined, the two hospitals handle approximately 700 surgeries and 1,100 hospitalizations per month. In the last year, respiratory problems accounted for 59.70% of all hospital admissions. 

The initial approach to pneumonias complicated by pleural effusion involved chest X-rays, followed by thoracic ultrasound examinations to estimate the volume and density of the pleural fluid and to identify the precise location of any debris or loculations. Chest CT scans were obtained to better visualize lung parenchymal involvement. CT scans were particularly used in order to identify, locate, and characterize loculations, pneumatoceles, pulmonary incarcerations, abscesses, pulmonary necrosis, and bronchopleural fistulas, especially in cases with persistent air leaks via water-seal drainage accompanied by pneumothorax, pneumomediastinum, or hydropneumothorax. 

Demographic and clinical data were recorded, including sex, age, symptoms, underlying diseases, indication for VATS, affected side of the chest, duration of prior closed thoracic drainage, timing of VATS after admission, culture results, postoperative chest tube duration, total length of hospital stay, complications, perioperative mortality, and outcomes. Perioperative mortality was defined as death within 30 days after surgery. 

All VATS procedures were performed in the operating room under general anesthesia with patients in the right or left lateral position. Either single-lung or dual-lung ventilation was employed, depending on the success of selective intubation. We used 12-, 10-, or 5-mm trocars for the insertion of 0° or 30° lenses, with one or two additional ports for 5-mm instruments. When necessary, intermittent carbon dioxide insufflation was used, maintaining a maximum pressure of 4-6 mmHg. 

For pleuropulmonary infections, the procedures consisted of lysis of adhesions; intermittent irrigation with warmed 0.9% saline solution; debridement; and removal of devitalized tissue, fibrinous exudates, and purulent secretions. Samples were collected for microbiological and histopathological analysis. In some instances, wedge resections were performed with a linear stapler. 

For statistical analysis, the data were analyzed with R software, version 4.4.1 (The R Foundation for Statistical Computing, Vienna,). Initially, descriptive analysis of categorical and continuous variables was performed. Categorical variables were presented as absolute frequencies and proportions, whereas continuous variables were described as mean, median, standard deviation, and minimum and maximum values, depending on the data distribution. 

The normality of continuous variables-length of hospital stay and length of closed drainage after VATS-was verified with the Shapiro-Wilk test. Given the violation of the normality assumption (p < 0.05), nonparametric tests were used for comparisons between independent groups. Therefore, differences between means were assessed with the Mann-Whitney U test. The significance level was set at 5% (p < 0.05) for all analyses. 

The present study was approved by the Research Ethics Committee of the *Instituto D’Or de Pesquisa e Ensino* (Protocol no. 90510125.3.0000.5249). 

## RESULTS

A total of 75 VATS procedures were performed during the study period. The annual distribution was as follows: 4 procedures in 2020, 4 in 2021, 22 in 2022, 29 in 2023, and 16 in 2024. The cohort consisted of 37 boys (51.4%) and 35 girls (48.6%), with a mean age of 50.2 months (range, 7 months to 17 years and 9 months). Of those, 22.7% were under two years of age, while most (72.1%) were under five. 

In only eight of the children (10.6%), the indications were unrelated to pleuropulmonary infections: two underwent lobectomies (for a recurrent abscess and congenital lobar emphysema); two underwent pleurodesis (for recurrent pneumothorax); one underwent VATS for correction of a thoracic deformity (pectus excavatum); two underwent VATS for biopsies of mediastinal tumors (later identified as ganglioneuroma and mature teratoma); and one underwent VATS for a paratracheal lymph node biopsy. 

Of the patients with pleuropulmonary infections, a chest CT scan was performed on all but one (1.4%), who could not undergo the scan because of severe respiratory failure requiring mechanical ventilation and associated hemodynamic instability. At least one thoracic ultrasound examination was performed for every patient. 

A total of 67 procedures were performed for complicated pneumonias, 46 of which involved empyema. The interventions included the following: irrigation; lysis of adhesions; drainage of loculated empyema; drainage of abscesses; wedge resection or stump closure for alveolar or bronchopleural fistulas; decortication of a trapped lung; and bullectomy of pneumatoceles. 

Primary VATS, without prior closed thoracic drainage, was performed in 12 of the 46 procedures for empyema (26.0%). Of those 12, 8 (66.6%) were performed within 7 days of admission, and 4 (33.3%) were performed after the eighth day of hospitalization because of a lack of clinical response to antibiotics. In 34 of the 46 cases of empyema (73.9%), VATS was performed because of clinical failure after antibiotics and closed drainage. In 21 of the 67 pleuropulmonary infections (31.3%), VATS was performed to manage abscesses, persistent bronchopleural fistula, encysted pneumothorax, or a trapped lung. 

Excluding the eight patients with noninfectious indications, the average chest drainage duration after the 18 VATS procedures performed within 7 days of admission was 13.3 days. For the 49 VATS procedures performed 8 or more days after admission, the average postoperative drainage duration was 12.8 days (Mann-Whitney test, p = 0.960). The mean total hospital stay was significantly longer in the group that underwent VATS after 8 days (38.0 days vs. 28.3 days; Mann-Whitney test, p = 0.002; Table 1). 

**Table 1:** Between-group comparison of the time to video-assisted thoracoscopic surgery.

Variable	Group	N	Mean	Median	SD	p*
Length of hospital stay	≤ 7 days	18	28.3	27.0	8.36	0.002
	≥ 8 days	49	38.0	35.0	10.81	
Closed drainage after VATS	≤ 7 days	18	13.3	11.0	8.66	0.960
	≥ 8 days	49	12.8	11.0	7.72	

VATS: video-assisted thoracoscopic surgery. *Mann-Whitney test for independent samples. Significant differences were considered for p < 0.05.

Of the 46 patients initially identified with empyema, one was excluded from the analysis because of an atypical length of stay (101 days) for marrow aplasia, leaving 45 patients for analysis. Most of the procedures were performed late (≥ 8 days after admission), corresponding to 73.3% of cases, whereas 26.7% were considered early (≤ 7 days). In the empyema cases, the mean age was 50.2 months (SD = 33.2; median = 43), with a range of 9-168 months. The mean length of hospital stay was 33.6 days (SD = 10.2; median = 32). The interval between admission and closed drainage averaged 4.9 days (SD = 6.05; median = 3.5), whereas the drain retention time after VATS averaged 12.2 days (SD = 7.9; median = 10), ranging from 4 days to 49 days.

A comparison of the time to VATS between the two groups (≤ 7 days and ≥ 8 days) showed that the time to closed drainage after VATS was similar, with no significant difference (p = 0.511). Conversely, the total hospital stay was significantly shorter in patients undergoing early VATS (median, 24.5 days) than in those undergoing VATS later (median, 34 days; p < 0.001; Table 2). 

**Table 2:** Comparison of clinical variables according to the time to video-assisted thoracoscopic surgery in patients with empyema (n = 45).

Variable	Group	N	Mean	Median	SD	SE	p*
Closed drainage after VATS, days	≤ 7 days	12	10.8	9.5	5.59	1.61	0.511
	≥ 8 days	33	12.8	10.0	8.60	1.50	
Length of hospital stay, days	≤ 7 days	12	25.5	24.5	6.52	1.88	< 0.001
	≥ 8 days	33	36.6	34.0	9.69	1.69	

VATS: video-assisted thoracoscopic surgery. *Mann-Whitney test.

Bacterial growth was found in 7 (14.9%) of 47 cultures of samples obtained during VATS, as follows: *Streptococcus pneumoniae* (in 4); *Stenotrophomonas maltophilia* (in 1); *Streptococcus viridans* (in 1); and *Pseudomonas aeruginosa* (in 1). Blood product transfusion was required in 17 of 46 patients (36.9%). 

Complications related to surgery occurred in 16 of 75 procedures (21.3%), with greater-than-usual bleeding in 5, subcutaneous emphysema in 4, and hemodynamic instability in 3. Less common complications occurring in only one patient each were as follows: pneumothorax; persistent bronchopleural fistula; contralateral lung atelectasis; and hemoptysis. In three children, the bronchopleural fistula persisted despite VATS. No VATS procedure was converted to open thoracotomy. 

In our cohort of 75 patients, there was one death, which was not directly related to the VATS procedure. 

## DISCUSSION

VATS is applicable for diagnosing and treating a broad spectrum of pediatric conditions,^7^ ^,^ ^11^ a fact reflected in the substantial volume of recent literature on the topic. 

In our series, only 8 procedures were performed for indications unrelated to pleuropulmonary infections. These were elective surgeries conducted by a dedicated thoracic surgery team. Globally, the applications of thoracoscopy have expanded significantly from its initial use for pleural debridement in empyema to lately include more complex procedures such as mediastinal tumor resection, repair of diaphragmatic defects, and correction of esophageal atresia.^11^ ^,^ ^12^ Although endoscopic surgery involves a steep learning curve, our pediatric surgical team has accrued considerable experience, leading to a marked increase in minimally invasive surgery for empyema debridement since 2020. 

In our cohort, the indications for VATS in the context of infection were categorized as follows: 12 procedures were performed as a primary intervention for pleural sepsis prior to chest tube insertion; 34 were undertaken because of persistent symptoms despite antibiotic therapy and closed thoracic drainage; and 21 were indicated to manage complications of necrotizing pneumonia. Although some studies have advocated for early intervention,^13^ our sample of patients undergoing VATS before the fifth day of hospitalization was very small. 

When comparing postoperative outcomes, we found that the duration of chest tube drainage was similar between children undergoing VATS before and after the seventh day of hospitalization. However, the early VATS group experienced a significantly shorter total hospital stay, a finding that is consistent with those of another retrospective study.^13^

Our institutional protocol often involves initial thoracentesis for fluid analysis, followed by chest tube insertion if empyema is identified. Consequently, VATS is typically reserved for cases in which this conservative management fails. It is important to note that the present study did not capture the failure rate of conservative therapy or the hospital stay of nonsurgical patients, given that our analysis was restricted to children who underwent VATS. 

The management of pediatric empyema often involves choosing between fibrinolytic therapy and VATS, with both strategies offering distinct clinical and economic profiles. One randomized multicenter trial found that both approaches yielded comparable outcomes in terms of hospital stay and recovery, although the need for a second intervention was marginally higher in the VATS group (15%) than in the fibrinolytic group (10%).^14^ ^,^ ^15^

Fibrinolytic therapy is generally less costly than VATS.^16^ This cost differential, coupled with similar efficacy, has led some to question the role of VATS as a first-line treatment, suggesting it may be better reserved for cases refractory to fibrinolytics.^15^ In our health care setting, the remuneration model precludes the use of fibrinolytic therapy for empyema. It was employed in only one case involving a child of Jehovah’s Witness parents to avoid surgical blood reserve; however, that patient ultimately required VATS because of lung entrapment. 

Although the literature on adult patients suggests that residual pleural thickening should be addressed with decortication to prevent restrictive lung disease, long-term follow-up in children managed nonoperatively with antibiotics and drainage has shown pulmonary function tests within normal limits.^17^

As with any surgical procedure, VATS in children carries a risk of complications that can impact the clinical course. The nature and severity of such complications vary, necessitating meticulous surgical planning and technique. Reported complications include accidental lobar bronchus transection, pneumothorax, hemorrhage, persistent air leak, iatrogenic phrenic nerve injury, and surgical site infection.^10^ ^,^ ^18^

Despite the aforementioned risks, VATS remains a valuable technique in pediatric surgery, characterized by a low mortality rate and a generally favorable safety profile. Our complication rate was 21.3%, with hemorrhage and subcutaneous emphysema being the most common. There were no procedure-related deaths. Ongoing advances in technique and instrumentation are expected to further mitigate these risks.^11^ ^,^ ^19^

Notably, only 4 of 75 patients (5.3%) were under 12 months of age. Two of those infants experienced postoperative complications, namely, contralateral lung atelectasis and subcutaneous emphysema. Although younger children are more vulnerable to intraoperative challenges such as hypercarbia and oxygen desaturation,^9^ these were not observed in our infants. 

Conversion to thoracotomy may be necessary because of complications or technical difficulties, with reported rates in children varying widely from 0% to 33%.^20^ This variability is influenced by surgical indication, procedural complexity, and surgeon experience. One study comparing an earlier (2005-2009) and a later (2010-2011) cohort found that conversion rates dropped from 27% to 8%, underscoring the impact of the learning curve.^20^ In our series, no conversions to thoracotomy were required, although it is relevant that we performed only two VATS lobectomies. 

In conclusion, our experience demonstrates that VATS can be performed safely in pediatric patients, with a low risk of serious complications and conversion to open surgery. It is a highly effective modality for managing a wide range of thoracic conditions in children. In agreement with other studies, our study found that the total length of hospital stay was shorter in children undergoing VATS in the first week after admission. 
